# The Role of Inflammatory Cytokines, the RANKL/OPG Axis, and the Immunoskeletal Interface in Physiological Bone Turnover and Osteoporosis

**DOI:** 10.1155/2013/125705

**Published:** 2013-02-03

**Authors:** M. Neale Weitzmann

**Affiliations:** ^1^Atlanta Department of Veterans Affairs Medical Center, Decatur, GA 30033, USA; ^2^Division of Endocrinology and Metabolism and Lipids, Department of Medicine, Emory University School of Medicine, 101 Woodruff Circle, 1305 WMRB, Atlanta, GA 30322, USA

## Abstract

Although it has long been recognized that inflammation, a consequence of immune-driven processes, significantly impacts bone turnover, the degree of centralization of skeletal and immune functions has begun to be dissected only recently. It is now recognized that formation of osteoclasts, the bone resorbing cells of the body, is centered on the key osteoclastogenic cytokine, receptor activator of NF-**κ**B ligand (RANKL). Although numerous inflammatory cytokines are now recognized to promote osteoclast formation and skeletal degradation, with just a few exceptions, RANKL is now considered to be the final downstream effector cytokine that drives osteoclastogenesis and regulates osteoclastic bone resorption. The biological activity of RANKL is moderated by its physiological decoy receptor, osteoprotegerin (OPG). New discoveries concerning the sources and regulation of RANKL and OPG in physiological bone turnover as well as under pathological (osteoporotic) conditions continue to be made, opening a window to the complex regulatory processes that control skeletal integrity and the depth of integration of the skeleton within the immune response. This paper will examine the interconnection between bone turnover and the immune system and the implications thereof for physiological and pathological bone turnover.

## 1. Introduction

Mineralized bone consists of a protein matrix comprising predominantly, but not exclusively, collagen type I fibers that are layered down in oriented linear bundles. This protein matrix scaffold is coated with a layer of mineral, predominantly calcium phosphate in the form of crystals of hydroxyapatite [1].

The skeleton forms in early life, mainly through endochondral ossification in which bone is initially patterned in mineralized cartilage followed by replacement of the cartilage template by mineralized bone. Some skeletal components including certain bones of the skull such as the calvaria are formed without a cartilage intermediate through direct matrix and mineralization deposition, a process referred to as intramembranous ossification [2].

The skeleton achieves its final shape and ultimate form through bone modeling, a process involving the coordinated activity of bone synthesizing osteoblasts and bone resorbing osteoclasts. This process of selective bone deposition and removal sculpts the skeleton to achieve final shape and optimal load bearing capacity [3, 4]. Bone modeling continues until early adulthood in humans at which time peak bone mineral density (BMD) and bone size are achieved. Thereafter, the skeleton undergoes a process of bone renewal referred to as remodeling. In remodeling bone resorbing osteoclasts and bone forming osteoblasts work in unison to resorb damaged or worn bones and resynthesize new bones, as well as allowing for growth and expansion of the marrow cavity and increase in trabecular thickness. Initially, the process of bone remodeling is homeostatic, as the rate of osteoclastic bone resorption is matched by the rate of osteoblastic bone formation. The net result is bone repair without net gain or loss of bone mass, and in principle bone remodeling has the capacity to replace approximately 25 percent of trabecular bone and 3% of cortical bone each year [3, 4].

In reality, this capacity of the skeleton to regenerate in a homeostatic manner is short-lived, and both men and women start losing bone by their fourth decade of life. After menopause bone loss is greatly intensified for a time in women where a rapid phase of bone loss ensues for 5–10 years as a consequence of estrogen decline. Men experience a slower linear loss of bone mass as a consequence of a progressive decline in sex steroids [3].

This persistent loss of bone density and mass with age leads to osteopenia (low bone mass) and ultimately culminates in osteoporosis, a condition that greatly increases the risk of a bone fracture. Although younger males (between the ages of 15 to 49) are almost 3 times more likely to sustain a fracture than females [5], in older populations fracture incidence is significantly higher in women than men where fragility fractures affect almost one in two older women compared to one in three to four older men [6]. The reason for increased fracture incidence in women is complex but in addition to losing bone faster following the menopause, women also accumulate less skeletal mass than men during growth, particularly in puberty. This results in smaller bones with thinner cortices and with a smaller diameter that have a relatively reduced load bearing capacity [3].

The consequences of fracture can be devastating and are a significant cause of morbidity and mortality [7, 8]. Vertebral fractures can cause severe back pain and disability [9], while hip fractures are usually extremely serious and nearly always result in hospitalization and major surgery. Hip fractures are fatal about 20% of the time and produce permanent disability about half the time [8, 10]. In some studies one-year mortality after hip fracture has been seen to reach as high as 30% [11]. Survivors often suffer tremendous indignity and decline in quality of life due to the need for extensive rehabilitation and protracted nursing home stays that often deprive the aged of their mobility and independence, sometimes permanently [12]. The financial consequences for patients and society of bone fractures are considerable [12]. Total fractures in 2005 were >2 million costing nearly $17 billion, and these costs are projected to rise by almost 50% by 2025 [13].

There have been tremendous advances over the last 2 decades in understanding the processes that regulate physiological bone turnover and in delineating the mechanisms by which multiple pathological conditions collide with the skeleton. A surprisingly large number of these maladies center on inflammation and immune dysregulation associated with natural aging and leading to collateral damage to the skeleton. Furthermore, infections and immunological diseases such as human immunodeficiency virus-1 (HIV-1) and infection/acquired immunodeficiency syndrome (AIDS) are now recognized to take a severe toll on the skeleton, predisposing even relatively young men and women to the consequences of bone fracture. 

The immunoskeletal interface involves the centralization of immune and skeletal functions around common cell types and cytokine effectors that control physiological bone mass, but lead to skeleton deterioration under states of prolonged immune activation. Realization of this interconnection of the immune and skeletal systems more than a decade ago has now spawned the emergence of a dedicated field of study, sometimes referred to as ‘‘osteoimmunology,” different aspects of which have been the subject of a number of excellent review articles including [14–23].

## 2. Osteoclasts and the Receptor Activator of NF-*κ*B (RANK)/RANK Ligand (RANKL)/Osteoprotegerin (OPG) System

Osteoclasts are the unique cells of the body tasked with resorbing bone. These giant multinucleated cells bind down onto bone surfaces creating a sealing zone and a ruffled-membrane border into which apical proton pumps accumulate hydrogen ions that combine with chloride ions to form hydrochloric acid, which degrades bone mineral. The exposed collagen matrix is attacked by acid resistant endosomal and lysosomal enzymes that cleave collagen fibers effectively removing small quantities of bone [24].

A central enigma in the world of osteoclast biology has been how osteoclasts form and what regulates their differentiation and activity. It has long been recognized that the osteoclast precursors circulate amid the monocyte/macrophage population and differentiate into preosteoclasts that fuse to form giant bone resorbing mature osteoclasts [25]. It was further recognized that inflammatory processes such as rheumatoid arthritis and periodontal infection predispose to osteoclastic bone loss, giving rise to speculation that inflammatory cytokines such as Interleukin (IL) −1, tumor necrosis factor alpha (TNF*α*), and IL-6 may play critical roles in osteoclast formation [26, 27]. 

Interestingly, these cytokines when placed on purified monocytes fail to induce significant osteoclast formation suggesting an indirect mechanism of action. Because bone formation and resorption work in unison (are coupled) during bone modeling and remodeling, it was further speculated that osteoblasts may regulate the activity of osteoclasts. Studies from the Suda group in Japan were indeed able to demonstrate that purified osteoblasts treated with vitamin D and cocultured in contact with osteoclast precursors (monocytes) from mouse spleen promoted significant formation of multinucleated cells capable of resorbing pits on dentin, a bone substitute, and hence meeting the functional requirement for designation as osteoclasts [28].

However, the identity of the osteoclastogenic cytokine responsible for osteoclast formation and commonly referred to as osteoclast differentiation factor (ODF) would remain obscure for another decade.

By the late 1980s the technologies of molecular biology and protein biochemistry had evolved sufficiently to set the stage for a series of pivotal discoveries in the field of bone biology. The first, in 1997, was the identification, by two independent groups, of a potent negative inhibitor of osteoclastogenesis named osteoprotegerin (OPG) [29] by Amgen investigators in the USA and osteoclastogenesis inhibitory factor (OCIF) [30] by investigators in Japan. The following year, using advanced molecular biology approaches, the Amgen group was able to purify and define a ligand to OPG (OPG ligand (OPGL)) that potently stimulated the production of osteoclasts from purified precursors [31]. Almost simultaneously, using an extensive biochemical purification strategy, the Suda group in Japan identified an equivalent ligand to OCIF on the surface of osteoblasts whose potent osteoclastogenic activity they ascribed to the elusive ODF [32]. OPGL and ODF were found to be structurally and functionally identical, potently promoting osteoclast formation in the absence of any other exogenous factors, with the exception of the monocytic survival factor macrophage colony stimulating factor (M-CSF). Interestingly, a factor structurally identical to OPGL and ODF had been previously reported by two independent groups of immunologists and had been named receptor activator of NF-*κ*B ligand (RANKL) [33] and tumor necrosis factor-related activation-induced cytokine (TRANCE) [34]. These groups showed that this factor, expressed by T cells, had immunomodulatory functions through receptors (RANK/TRANCE-receptor) present on dendritic cells [33–35]. Although all these different terms were used interchangeably for some years, in the context of bone biology the preferred terms are now RANKL to describe the osteoclastogenic cytokine, OPG to describe its inhibitor, and RANK to describe the receptor for RANKL.

It is now clear that RANKL is the key final effector osteoclastogenic cytokine and in the presence of a permissive concentration of M-CSF is capable of inducing osteoclast formation and promoting osteoclast resorptive activity in the absence of any other cytokines. OPG functions as a decoy receptor, preventing association of RANKL with RANK receptor [35] and thus moderating osteoclastogenesis and bone resorption (reviewed in [25, 36, 37]). 

It is now understood that the osteoclast precursor is a cell expressing one or more markers of the monocyte lineage (typically CD14, cFms (the M-CSF receptor), or CD11b) and RANK. Binding of RANKL to RANK initiates a sequence of signal transduction pathways mediated though TNF receptor-associated factor 6 (TRAF6) and including NF-*κ*B [38–40], c-Jun N-terminal kinase (JNK)/cJun/fos [38, 41–43], and nuclear factor of activated T cells (NFAT) [44–46] that initiate the differentiation of the early osteoclast precursor into a preosteoclast (recognized *in vitro* or *ex vivo* by the expression of the enzyme tartrate resistant acid phosphatase (TRAP)). Preosteoclasts ultimately fuse with each other into mature multinucleated bone resorbing osteoclasts recognized by the expression of key osteoclast markers including TRAP [47], calcitonin receptors [28], cathepsin K [48], pp60c-src [49], matrix metalloproteinase 9 (MMP9) [50], and the alpha V beta 3 integrin chains [51, 52]. Osteoclasts originating from multiple species can further be identified by reactivity to a unique osteoclast-specific monoclonal antibody (121F) [53].

The central role of RANKL in basal osteoclast formation and bone homeostasis has been established in animal models where genetic deletion of RANKL leads to an osteopetrotic phenotype characterized by a complete absence of osteoclasts and a failure of tooth eruption (an osteoclast driven process) [54]. Similarly, the key physiological role of OPG has been established in knock-out (KO) and transgenic overexpression studies in mice where deletion of the OPG gene leads to large numbers of osteoclasts and to severe bone erosions [54, 55]. By contrast transgenic overexpression causes an osteopetrotic phonotype in mice [56].

Consistent with the animal studies activating mutations in the receptor RANK are associated with familial expansile osteolysis, a rare autosomal dominant bone disorder characterized by focal areas of increased bone remodeling [57]. Many forms of Paget's and Juvenile Paget's disease may result from deletions/mutations in the OPG gene (*TNFRSF11B*) [58–60].

Human disease associated with mutations in the RANKL gene is rare, although a novel mutation in the gene for transforming growth factor beta 1 (*TGF*β*1*) and a missense change in *TNFSF11* encoding RANKL may both contribute to the bone phenotype associated with Camurati-Engelmann disease [61].

From a therapeutic standpoint several studies have demonstrated the capacity of OPG suppression to ameliorate osteoporotic states and/or improve bone mass in ovariectomized animals (a model of postmenopausal osteoporosis) by means of adenoviral OPG delivery in mice [62], a single intravenous OPG injection in ovariectomized rats [63], weekly treatment of gonadal intact monkeys [64], and a single injection in postmenopausal women [65]. Although OPG has never been successfully translated into an approved therapy for humans, a humanized neutralizing antibody (Denosumab) directed against RANKL is now US Food and Drug Administration (FDA) approved for fracture prevention and amelioration of postmenopausal and other forms of osteoporosis [66, 67].

The evidence is now extremely strong supporting a final effector role of the RANK/RANKL/OPG system in osteoclast formation and the regulation of bone resorption. It is also now clear that inflammatory cytokines such as IL-1, TNF*α* and M-CSF that have long been associated with osteoclastic bone loss, function by promoting RANKL production by osteoblast precursors (bone marrow stromal cells (BMSC)) and/or mature osteoblasts [68, 69]; and/or by reducing OPG production [70], and/or by upregulating the receptor RANK on osteoclast precursors [71] thus increasing their sensitivity to prevailing RANKL concentrations. IL-1 and TNF*α* have long been implicated in osteoclast formation in postmenopausal osteoporosis [26, 72] and in animal models thereof (ovariectomy) [73–76]. In a recent study it was further demonstrated that IL-1 mediates the osteoclastogenic effect of TNF*α* by enhancing stromal cell expression of RANKL and directly stimulating differentiation of osteoclast precursors [69]. TNF*α* turns out to have another property that is relatively unique among the inflammatory cytokines; TNF*α* has particularly potent effects on osteoclastogenesis as it not only promotes RANKL production [68] but synergizes with RANKL to amplify osteoclastogenesis [77, 78] and to intensify osteoclastic resorption [79] by integrating with RANKL-induced signal transduction pathways [79, 80]. These effects are likely a consequence of the fact that RANKL is in fact a TNF-superfamily member and functions through many of the same pathways induced by TNF*α* itself. Although some early studies suggested that TNF*α* may be capable of direct RANKL-independent osteoclastogenesis [81], the weight of evidence now goes against this theory, and it is likely, given the ability of RANKL to amplify RANKL-induced osteoclastogenesis, that in these studies the mouse-derived osteoclast precursors were endogenously exposed to RANKL *in vivo* and hence were primed to respond to TNF*α* *ex vivo*.

As a consequence of the inflammatory action of TNF*α*, its overexpression *in vivo*, as in the case in transgenic mice, leads to inflammation resembling human rheumatoid arthritis with significant focal and systemic bone erosions [82, 83]. Indeed TNF*α* decoy receptors are now accepted therapies for human rheumatoid arthritis [84].

## 3. TNF*α* and the NF-*κ*B Signal Transduction Pathway in Bone Resorption

The molecular action of TNF*α* is in large measure a consequence of its ability to potently stimulate activation of the NF-*κ*B transcription factor. This pathway is also a major mediator of RANKL-induced signal transduction, and not surprisingly TNF*α* potently augments RANKL-induced osteoclast formation. The importance of NF-*κ*B to osteoclast development, function, and survival has long been recognized [41, 85–88] and is strongly supported by evidence of osteopetrosis in p50 and p52 NF-*κ*B double KO mice, as a consequence of defective osteoclast differentiation [89]. The NF-*κ*B subunit RelB has further been shown to play a key role in osteoclast differentiation [87] while the p65 NF-*κ*B subunit prevents JNK-mediated apoptosis during RANKL-induced commitment to the osteoclast phenotype [41]. Pharmacological antagonists of NF-*κ*B signaling have been shown to suppress osteoclast activation [86] and to blunt osteoclastic bone resorption and ameliorate bone loss induced by ovariectomy in mice [90], multiple myeloma induced osteoclast formation and activity *in vitro* [91], implant particle-induced osteoclastogenesis [92], and in a rheumatoid arthritis animal model *in vivo* [93].

## 4. NF-*κ*B and Bone Formation

Surprisingly, when it comes to osteoblasts and bone formation NF-*κ*B signal transduction is quite dissimilar to that of osteoclast formation/bone resorption and is in fact potently inhibitory. It has long been recognized that the p65 NF-*κ*B subunit is inhibitory to vitamin D-stimulated osteoblastic osteocalcin transcription (a measure of osteoblast differentiation) [94]. As a potent stimulator of NF-*κ*B, it is not surprising that several studies have documented inhibitory effects of TNF*α* on osteoblast formation [95, 96] while in a mouse fracture healing model the pharmacological suppression of TNF*α* reverses age-related defects in bone formation [97]. 

Mechanistically, one mechanism by which TNF*α* suppresses osteoblast differentiation is by suppressing Smad signaling in differentiating osteoblasts, through an NF-*κ*B-mediated process [98]. Smad signals are mediated in part by members of the transforming growth factor beta (TGF*β*) superfamily, including TGF*β* itself and bone morphogenetic proteins (BMPs). TGF*β* and BMPs are key factors needed for directing the commitment of osteoblast precursors, along an osteoblastic trajectory and for their differentiation into mineralizing osteoblasts [99, 100]. BMPs are potent physiological inducers of osteoblast differentiation and angiogenesis [101], and BMP-2 is now employed as a pharmacological tool for promoting bone repair in orthopaedic implants [102].

TGF*β* is thought to play important roles in early osteoblast lineage commitment [103] and in the recruitment of bone marrow stromal cells to bone resorptive sites, through a Smad-dependent signaling pathway [104]. TGF*β* regulates osteoblast differentiation in a biphasic manner as TGF*β* stimulates development and proliferation of early osteoblasts, but inhibits their maturation. How TGF*β* and BMPs coordinate osteoblast differentiation is unclear. TGF*β* strongly stimulates the synthesis of extracellular matrix proteins [105] but *in vitro* studies show an inhibitory effect on the final mineralization process, which nonetheless does occur *in vivo* despite the presence in the bone microenvironment of high concentrations of TGF*β* [106, 107]. One explanation for this apparent contradiction is that the TGF*β* receptor is downregulated during osteoblast differentiation following exposure of cells to collagen synthesized by the osteoblasts and enabling osteoblasts to escape from the inhibition of TGF*β* [108].

The interface between NF-*κ*B and Smad signaling is poorly understood but studies reveal that NF-*κ*B signaling upregulates the inhibitory Smad, Smad-7 that antagonizes osteoblast differentiation [109]. These data are consistent with studies showing that TNF*α* promotes systemic bone loss in part, by stimulating proteasomal degradation of BMP proteins by a mechanism involving Smad ubiquitination regulatory factor 1 (Smurf1) [110]. Furthermore, TNF*α* is reported to inhibit BMP-induced osteoblast differentiation through activating stress-associated protein kinase (SAPK)/JNK signaling [111].

Indeed, it has long been recognized that TNF*α* contributes to ovariectomy-induced bone loss not only by promoting bone resorption, but also by suppressing the compensatory response in bone formation that is initiated in response to bone resorption, a consequence of the homeostatic coupling process. Indeed, pharmacological suppression of TNF*α* increases bone formation in the context of ovariectomy [75]. Consistent with these data we have demonstrated that estrogen promotes bone anabolism *in vitro* by antagonizing NF-*κ*B activation in the osteoblastic cell line MC3T3 [112]. 

In contrast to inflammatory states characterized by high concentrations of TNF*α*, we recently investigated the effect of physiological endogenous levels of TNF*α* on basal bone formation *in vivo* using TNF*α* KO mice, as well as TNF*α* receptor type I (p55) and type II (p75) KO mice. Our studies demonstrated that mice null for TNF*α* and the p55 receptor exhibited a significantly elevated bone mineral density (BMD) and bone volume as a consequence of significantly elevated bone formation. Our data suggested that TNF*α* potently diminished the acquisition of peak bone mass demonstrating the powerful effect of even low concentrations of endogenous TNF*α* on the bone formation machinery [98]. Consistent with these effects an extensive *in vivo* investigation of NF-*κ*B signaling on basal bone formation has demonstrated that a conditional inactivation of NF-*κ*B signaling in osteoblasts by means of transgenic overexpression of the inhibitor of *κ*B kinase (IKK) increases trabecular bone mass and BMD and reverses ovariectomy-induced bone loss by enhancing bone formation [113].

Like the action of estrogen, a number of natural and nutritional agents that have been associated with bone protective effects by exhibiting stimulatory activities on bone formation and suppressive actions of bone resorption appear to exploit this divergent role of NF-*κ*B signaling on bone cells. We have shown that vitamin K2 [114], Honokiol [115], Zinc [116], and the carotenoid *p*-hydroxycinnamic [117, 118] are all capable of modulating NF-*κ*B activation in osteoblast and osteoclast precursors *in vitro* by antagonizing RANKL- and TNF*α*-induced NF-*κ*B activation in osteoblasts and osteoclasts, respectively.

## 5. RANKL and OPG Production under Physiological and Pathological Conditions

### 5.1. Physiological RANKL Production

Although it is recognized that many cell types have the capacity to produce RANKL and OPG, the major source of both of these factors, especially under basal conditions, has generally been ascribed to osteoblasts, the bone building cells, and their precursors, cells originating from mesenchymal stem cells (MSCs) and sometimes referred to as bone marrow stromal cells (BMSCs). These conclusions however, are primarily based on *in vitro* studies and are now contested by newly emerging *in vivo* data [119]. In a recent *in vivo* study targeted osteoblast deletion in mice was found to lead to osteopenia, as expected; however bone resorption continued normally in the absence of bone formation, and osteoclast number was not significantly impacted. This outcome suggested that osteoblasts and bone formation do not control the extent of bone resorption through production of balsa RANKL *in vivo *as previously believed [120].

This concept of osteoblast-independent sources of RANKL was further illustrated in two recent back-to-back studies in the journal Nature Medicine that have challenged our notions of where basal RANKL originates. Using a state-of-the-art Cre-directed conditional RANKL KO system, mice were generated with specific RANKL gene deletions in MSCs and revealed that RANKL expression in mesenchyme-derived cells was essential for osteoclast formation in growing long bones, but not in vertebrae, or calvaria. Furthermore, tooth eruption was normal in these mice suggesting that the source of RANKL necessary for that process is not MSC derived [119]. MSCs are pluripotent cells that give rise to multiple cell lineage including osteoblasts/osteocytes, chondrocytes, fibroblasts, myocytes, and adipocytes [121]. Because the MSC deletion potentially impacted multiple lineages, further specific deletions of RANKL were performed in late-proliferating chondrocytes, mature osteoblasts, and in osteocytes (terminally differentiated osteoblasts entombed in bone matrix). RANKL derived from hypertrophic chondrocytes was found to be essential for mineralized cartilage resorption (a process central to endochondral bone formation) while osteocytes were necessary for bone remodeling as well as for bone resorption associated with unloading, in a tail-suspension model [119].

Interestingly, although activated T and B cells are well-recognized sources of RANKL, conditional RANKL KO in T and B cells failed to impact bone mass suggesting that RANKL derived from lymphocytes is not involved in the regulation of basal bone remodeling [122]. In another study, purified bone cell cultures *in vitro* revealed that RANKL expression and osteoclast formation by osteocytes are significantly higher than those of osteoblasts and stromal cells. The importance of osteocytes as a source of RANKL was further confirmed using an osteocyte conditional RANKL KO model that revealed that the development of a significant osteopetrosis occurs in the absence of RANKL production by osteocytes [123] suggesting a critical role of osteocyte RANKL in basal bone modeling/remodeling. Taken together these studies reveal an essential role for RANKL expressed by osteocytes and chondrocytes in basal osteoclastogenesis.

#### 5.1.1. RANKL-Independent Osteoclastogenesis

Although there is no doubt as to the importance of the RANK/RANKL/OPG axis in physiological and pathological bone turnover, a number of cytokines have now been identified that directly promote osteoclastogenesis in a RANKL-independent manner and may contribute to bone loss associated with certain pathological conditions, including inflammation.

#### 5.1.2. Osteoclastogenic Factor of Activated T Cells (SOFAT)

In 2001 we reported the results of a series of studies examining the mechanisms by which activated T cells promote osteoclastogenesis in inflammatory conditions and the reasons for spontaneous osteoclast formation in cultures of peripheral blood mononuclear cells, a system that is devoid of stromal cells/osteoblasts or exogenous cytokines. These studies found that both CD4^+^ and CD8^+^ T cells, when activated by the lectin phytohemagglutinin-P (PHA), potently stimulate human osteoclast formation *in vitro*. Following the discovery of RANKL as the key osteoclastogenic factor we were in fact able to demonstrate that RANKL production was clearly responsible for a significant proportion of the osteoclastogenic activity of the activated T cells. Interestingly, we also documented an intriguing residual osteoclastogenic activity that persisted in the presence of saturating concentrations of OPG and the RANKL inhibitor and that accounted for approximately 30% of the T-cell-dependent effect [124]. Similar data were found for IL-7-induced T-cell-mediated osteoclast formation in culture [125]. We further demonstrated that activated T-cell-conditioned medium had the ability to superinduce osteoclastogenesis in cultures of purified monocytes maximally stimulated by saturating concentrations of RANKL and M-CSF [124]. These data led us to conclude that activated T cells are able to promote osteoclastogenesis via both RANKL-dependent and RANKL-independent mechanisms [124, 125].

It was almost a decade before the exact nature of this controversial RANKL-independent osteoclastogenic cytokine was delineated. Using an intensive biochemical purification strategy we were able to fractionate conditioned medium from human activated T cells by high-performance liquid chromatography (HPLC) and recover biologically active fractions for further resolution by sodium dodecyl sulfate (SDS), and polyacrylamide gel electrophoresis (PAGE) which revealed a major candidate protein that was further characterized by mass spectrometry. With its protein sequence identified the putative factor was cloned and expressed using recombinant DNA technologies. The resulting protein which we termed secreted osteoclastogenic factor of activated T cells (SOFAT) was found to derive from an unusual mRNA splice variant coded by the threonine synthase-like 2 gene (*THNSL-2*) homolog. What was particularly surprising was that *THNSL-2* is a remnant of a gene that codes for threonine synthase in plants and microorganisms but is nonfunctional in higher organisms. Despite this lack of function *THNSL-2* has remained remarkably conserved in mammalian species, suggesting an ongoing functionality. Although the physiological/pathological function of SOFAT remains unknown, recombinant SOFAT was capable of stimulating functional osteoclast formation in the absence of osteoblasts or exogenous RANKL and in a manner that was completely insensitive to saturating concentrations of the RANKL inhibitor osteoprotegerin. Like RANKL, however, SOFATs osteoclastogenic activity was potently amplified by the inflammatory cytokine TNF*α* [126].

SOFAT had a further function, the capacity to promote IL-6 production from osteoblasts, another activity of formerly unknown source that had been previously documented in activated T-cell-conditioned medium [127].

While the function of SOFAT and its mechanisms of action remain to be characterized, we speculate that SOFAT may act to exacerbate inflammation and/or bone turnover under inflammatory conditions when T cells are activated such as in rheumatoid arthritis, periodontitis, and in conditions of estrogen deficiency, inflammatory disease states that are discussed in detail below.

#### 5.1.3. Homologous to Lymphotoxins Exhibiting Inducible Expression and Competing with Herpes Simplex Virus Glycoprotein D for Herpesvirus Entry Mediator (HVEM), a Receptor Expressed by T Lymphocytes (LIGHT)

Homologous to lymphotoxins exhibiting inducible expression and competing with herpes simplex virus glycoprotein D for herpesvirus entry mediator (HVEM), a receptor expressed by T lymphocytes (LIGHT), a member of the TNF superfamily (*TNFSF14*), that is significantly upregulated in the serum of human rheumatoid arthritis patients, is another cytokine expressed by activated T cells as well as monocytes, granulocytes, and immature dendritic cells. LIGHT has the capacity to induce osteoclast formation from both human and murine monocytes in the absence of RANKL and in a manner insensitive to OPG and RANK-Fc (a synthetic soluble decoy receptor of RANKL based on the RANKL receptor). Osteoclastogenesis was however impeded by blocking antibodies to the p75 TNF receptor. Like SOFAT, LIGHT may play an important role in supporting bone loss in patients with rheumatoid arthritis exacerbating RANKL-induced localized or systemic bone loss [128].

#### 5.1.4. TGF*β* in Osteoclastic Bone Resorption

Although TGF*β* has been shown to stimulate early bone formation (see the aforementioned), several studies have shown a capacity of this cytokine to also promote vitamin D-dependent production of osteoclasts in osteoblast cocultures [129] and to augment *in vitro* RANKL-mediated osteoclast formation [130, 131]. In contrast, TGF*β* has also been reported to inhibit osteoclastogenesis [132–134]. These confusing activities may be a consequence of biphasic effects whereby TGF*β* stimulates osteoclastogenesis at low doses, but suppresses it at high concentrations [129]. Potential mechanisms of osteoclast inhibition are through production of OPG [135] as well as suppression of RANKL from osteoblasts [136]. It has further been reported that estrogen promotes apoptosis of murine osteoclasts through production of TGF*β* [133]. Consistent with this apoptotic capacity we have reported that B cells *in vitro* are inhibitory to osteoclastogenesis as they secrete significant concentrations of TGF*β*, leading to apoptosis of osteoclasts and their precursors [134]. This may be further compounded by B cell OPG secretion, potentially amplified, in part, by a cell autonomous effect of TGF*β* on B cells.

Under conditions of estrogen deficiency in mice *in vivo* we have further shown that a decline in TGF*β* production is permissive for T-cell-driven inflammatory bone loss, as TGF*β* is a potent inhibitor of T-cell activation. Consequently, estrogen-induced TGF*β* may act to protect the skeleton from the ravages of inflammation [137] (discussed in detail later).

Interestingly, one study has reported direct TGF*β*-induced osteoclast formation by a RANKL-independent mechanism [138]. In this study addition of TGF*β* to cultures of human monocytes and RAW 264.7 cells (a monocytic cell line that acts as early osteoclast precursors) in the presence of M-CSF but in the absence of RANKL or other inflammatory cytokines induced the formation of functional bone resorbing osteoclasts. Resorption was not suppressed by OPG or by neutralizing antibodies to TNF*α*, its receptors, or the gp130 chain of IL-6, suggesting that TGF*β* acted independently of RANKL, TNF*α*, or IL-6 production. Furthermore, while RANKL stimulates large osteoclasts, TGF*β* induced large numbers of mononucleated preosteoclasts and small mature osteoclasts producing many small resorption lacunae [138].

The significance of TGF*β* in the regulation of basal osteoclastogenesis *in vivo* remains unclear with the potential for RANKL-dependent and -independent effects and direct inhibitory effects as well as indirect inhibitory effects through modulation of RANKL and OPG and through suppression of T-cell activation, creating a complex interplay.

### 5.2. Physiological OPG Production

Like RANKL, based on *in vitro* studies OPG production has traditionally been ascribed to osteoblasts and BMSCs. However, as far back as 1998 it was recognized that human B cells are a significant source of OPG and that modulation of the CD40 costimulatory receptor *in vivo* could markedly stimulate B cell OPG production [139]. These studies were considered of importance for the actions of RANKL in the context of immune function, but given the historical view that osteoblasts were the source of bone active OPG, the significance of these findings was overlooked in terms of a role in bone biology for almost a decade. In 2007 we reported that mice deficient in B cells had significantly elevated basal bone resorption and diminished BMD and volume. The central defect driving this imbalance in bone resorption relative to formation was a bone marrow deficiency in OPG, leading to an imbalance in the RANKL/OPG ratio that was permissive to bone resorption. Transplantation of B cells back into B cell KO mice was able to restore OPG to wild-type (WT) levels and rescue the development of osteoporosis [140].

Using WT mice we were able to quantify B-lineage sources of OPG production and were surprised to find that mature B cells accounted for 45% of total bone marrow OPG production. In addition, B cell precursors, immature B cells, and plasma cells further contributed OPG to the bone microenvironment with the entire B cell linage accounting for 64% of total OPG production [140].

An interesting finding was that as reported previously for human B cells [139], activation of the CD40 costimulatory receptor led to enhanced B cell OPG production. Because the cognate ligand of CD40 (CD40 ligand (CD40L)) is expressed by activated T cells, we further investigated the potential of T cells to regulate basal B cell OPG production using T-cell deficient nude mice, CD40L KO mice, and CD40 KO mice. These models confirmed that bone marrow OPG production is significantly compromised by T-cell deficiency and/or by genetic ablation of CD40 and CD40L costimulatory molecules, with a resulting increase in bone resorption and reduced bone mass [140]. The data were also consistent with a previous study demonstrating that depletion of CD4^+^ and CD8^+^ T lymphocytes in mice *in vivo* enhanced osteoclast formation *ex vivo* by a mechanism involving, in part, a pronounced suppression of OPG production [141].

Interestingly, osteopenia is a prominent clinical feature of patients with X-linked hyper-IgM syndrome, an inherited immune deficiency disorder caused by mutations in the gene encoding CD40L. Although the mechanisms for this bone loss are not clear, reduced interferon gamma (IFN*γ*) production has been reported by T cells [142]. OPG production was not investigated in this model.

Taken together these studies suggest that under basal conditions lymphocytes (both T cells and B cells) play an important stabilizing role and are protective of bone mass. As a consequence disruption of B cell or T cell function may have unfortunate repercussions for physiological bone turnover.

## 6. Consequences of Pathological Alterations to OPG Production

### 6.1. HIV-1 Infection and Osteoporosis

The quintessential immunodeficiency disease condition is the collapse of immunity that ensues following infection by the HIV-1 virus and leads to the constellation of pathological events generally referred to as acquired immunodeficiency syndrome (AIDS).

More than a decade ago alterations in skeletal metabolism were reported in AIDS patients on antiretroviral therapy (ART) [143]. The mechanisms responsible however were unclear but appeared to involve multiple underlying factors including ART, a collection of drugs used to suppress viral replication, traditional risk factors for osteoporosis such as smoking, drug use, and alcohol consumption, AIDS pathologies such as hypogonadism and renal disease, and a generalized low body mass index (BMI) that is often correlated with low bone mass [144–146]. In fact, based on this tight association between BMI and BMD, many now believe that low BMI associated with skeletal wasting is likely the most important underlying cause of low BMD in HIV patients [147]. Nonetheless, despite these clinical correlations no clear cause-effect relationship has been established between BMI and BMD in HIV infection, and, in fact, patients without wasting disease and with normal body weight also display reduced BMD at key sites including the lumbar spine and hip [148].

HIV infection itself is now considered a defined risk factor for osteoporosis although use of ART is recognized as an additional contributor to skeletal decline [149, 150]. Attesting to the impact of HIV infection recent studies show that an astounding two of every three patients with HIV infection, naive to ART, are osteopenic with 10% displaying outright osteoporosis [151].

Following initiation of ART, bone loss intensifies during the first 2 years of treatment leading to an additional 2%–6% decline in BMD, a decrease of a magnitude similar to that sustained during the first 2 years of menopause [150].

Until recently the consequences of this bone loss were unclear and the potential impact on patient health controversial. This, in part, was a consequence of the fact that the majority of HIV patients are relatively young and BMD is a poor indicator of fracture risk in younger populations (<55 years of age) [152]. Indeed, the standard clinical definitions of osteopenia and osteoporosis, as defined by the World Health Organization (WHO), are based on a statistical *T* score derived from BMD (quantified by means of dual-energy X-ray absorptiometry (DEXA or DXA) a bone densitometry technique). Osteopenia is defined as a *T* score of between −1.0 and −2.5 and osteoporosis as ≥−2.5. The *T* score represents the number of standard deviations (SDs) below a mean peak bone mass of an average healthy young adult derived from a reference database and typically further adjusted for race and gender [153]. While DEXA is a convenient and noninvasive technology, the sensitivity of BMD as a predictor of fracture risk is limited by the fact that this index does not take into account the geometrical and material characteristics of bone [154]. Furthermore, cancellous (trabecular) bone only represents 20% of total bone mass, and although both cortical and trabecular bone significantly contribute to structural strength of bone, DEXA provides an integrated measurement of both bone compartments significantly underestimating the contribution of trabecular bone. 

Despite fears being raised as to an epidemic of future fragility fractures as the mean age of the HIV demographic continues to trend upward [155] given the uncertainties in how to interpret changes in BMD in younger patients, physicians have been wary of intervening therapeutically in the absence of hard data regarding true fracture incidence. Over the last decade evidence of increased bone fracture has slowly accumulated to a critical mass. Among the first studies to report fragility fractures in HIV-infected patients on ART was from Guaraldi et al. who documented a 49-year-old male AIDS patient with osteopenia and a 51 year old male AIDS patient with osteoporosis, both of whom had suffered fractures after trivial trauma [156].

A central problem in investigating fracture in small cohorts is lack of statistical power owing to the relatively low rate of fracture in the general population and especially in men where fracture incidence remains low and stable (typically below 2%) until advanced age (>70 years of age) [157].

Studies involving ever-increasing population size have progressively cemented the concept that fracture incidence trends higher in HIV-infected populations. These studies include that of Arnsten et al., who studied BMD and incident fractures in 328 men over the age of 49 (mean age 55) with HIV infection and in 231 at risk individuals without HIV infection. BMD was reported to be significantly lower in HIV-infected compared with HIV-uninfected men at the femoral neck and lumbar spine after adjusting for age, weight, race, testosterone level, prednisone, and illicit drug use. Incident fracture rates per 100 person-years were 38% increase in HIV-infected men but fell short of being statistically significant, despite decreased BMD being associated with increased fracture risk [158]. 

In another fracture study involving a Canadian cohort, a ~2-fold higher fracture rate was observed in 138 HIV-infected women relative to 402 HIV^−^ controls. Interestingly the study reported that HIV^+^ women were more likely to have had fragility fractures despite BMD values that were not different than women from a national population-based cohort [159]. This study may again highlight the major weakness in DEXA in discriminating between BMD and bone quality, the latter being a more important index of load bearing capacity and fracture risk.

In 2008, results from a large population-based analysis of a total of 8525 HIV-infected and 2,208,792 non-HIV-infected patients confirmed previous fears regarding a fracture epidemic by demonstrating a significant escalation in fracture incidence in men beginning at relatively young age (40–49 years) and achieving a dramatic ~4 fold increase in HIV^+^ men by 60–69 years of age. In contrast to men, fracture incidence in healthy women is not stable and rises exponentially with age from around 0.5% at 30–39 years of age to ~3.5% by age 70–79. Importantly, HIV-infected women showed increased fracture prevalence relative to seronegative women at virtually every age and achieving an over 2-fold increase between 60 and 69 years of age [157].

This pivotal study has now been validated by other large population-based studies including the United States Department of Veterans Affairs (VA) cohort which comprised 39,375 HIV-infected patients and showed fracture rates of 24%–32% higher than uninfected populations [160] and in the HIV outpatient study (HOPS) study, a cohort of 5826 HIV patients where a 2- to 4-fold higher incidence of fracture was observed in the HIV seropositive population [161].

Not all studies have found increased fracture incidence however; for example, in the Women's Interagency HIV Study involving 1728 HIV-infected and 663 uninfected women the authors reported little difference in fracture incidence rates by HIV status in predominantly premenopausal women. Multivariate models did however reveal that race (white versus African-American), hepatitis C virus infection, and higher serum creatinine were statistically significant predictors of incident fracture [162].

While the bulk of recent data supports a significant decline in BMD and increase in facture incidence in HIV^+^ populations the mechanisms responsible remain unclear. One of the hallmarks of HIV-infection is the loss of CD4^+^ T cells as a consequence of a continuous stimulation of the immune system leading to activation-induced cell death (AICD) that is exacerbated by a failure to effectively restore T cells owing to reduced thymic function in the context of HIV infection [163]. Interestingly, although dramatic depletion of CD4^+^ T cells is one of the most recognized features of HIV infection, in fact the entire immune system is severely impacted with significant impairment of the B-cell compartment leading to compromised humoral immunity [164, 165]. Because T cells are central regulators of B-cell activity, alterations in T-cell function further drive impairment of B-cell activity. Alterations in the B-cell compartment include a significant decline in B-cell numbers as well as a significant increase in the frequency of immature/transitional B cells [165]. Although the exact causes of diminished B-cell number and alterations in B-cell subpopulations are poorly understood, cytokine imbalances, decreased T-cell function, direct viral exposure, and viral antigens are all likely to play a role [166].

### 6.2. B-Cell Disruption Drives Bone Loss in an Animal Model of HIV-1 Infection in the HIV-1 Transgenic Rat

The complexity of the human system and the limitations associated with undertaking invasive clinical studies has contributed in large measure to the confusion regarding the underlying causes of bone loss in HIV-1 infection and led us to exploit an animal model, the HIV-1 transgenic (Tg) rat, a model that is not confounded by lifestyle factors and effects of ART, to investigate the effects of HIV-1 on bone turnover.

Constitutive expression of HIV-1 viral proteins directed by a replication defective HIV-1 viral genome, integrated into the rat DNA, leads to a syndrome in these animals that significantly resembles the immunologic and clinical abnormalities observed in human AIDS [167]. Using this model we documented for the first time a severe defect in skeletal homeostasis that led to a significant decline in BMD and in bone volume. These alterations in skeletal mass were consistent with significantly elevated osteoclast numbers and bone resorption, a consequence of a significant decline in B-cell OPG production, compounded by a significant increase in B-cell production of RANKL. Production of RANKL is indeed an established property of activated B cells [168, 169] and of B-cell precursors [170]. This imbalance in the RANKL/OPG ratio was favorable to osteoclastic bone resorption and was likely further exacerbated by a dramatic increase in the number of osteoclast precursors [171].

These data link the disruptive impact of HIV infection on B-cell homeostasis to direct changes in bone turnover. In the context of humans, these alterations in the immunoskeletal interface are likely compounded by patient specific lifestyle factors and AIDS-associated pathologies thus contributing to the extremely high rates of bone disease exhibited by this population. Clinical studies to ratify these changes in humans are currently underway.

### 6.3. HIV, ART, and Inflammatory Bone Loss

While lymphocytes appear to play an important role in favor of the preservation of bone mass under physiological conditions, activated T and B cells, characteristic of inflammatory processes, are potent destroyers of bone and may cause skeletal degeneration in a wide range of pathological contexts.

Paradoxically, although HIV-1 infection leads to profound immunodeficiency, an inflammatory undercurrent is recognized to coexist in AIDS patients [22, 172] and is a strong predictor of disease progression [173]. Although the etiology is poorly defined, chronic immune activation is thought to result, in part, from persistent residual HIV-1 replication, chronic coinfections, and HIV-induced gastrointestinal mucosal damage that leads to internalization of high concentrations of bacterial metabolites and components including lipopolysaccharide (LPS), a bacterial cell wall protein that is potently immunogenic and leads to activation of both the innate and adaptive immune systems [173, 174]. LPS-induced inflammatory responses have long been recognized to stimulate production of RANKL, IL-1, and TNF*α* [175]. This inflammatory state in HIV-infected patients persists indefinitely even in the context of ART and may contribute to drive up bone resorption in AIDS patients even in the setting of effective viral suppression.

While ART has dramatically extended lifespan, troublesome metabolic complications including osteoporosis and elevated fracture prevalence are now undermining the quality of life of patients living on chronic ART [19]. Although HIV-1 infection and AIDS all contribute to severe skeletal deterioration, ART itself has long been recognized to independently contribute to bone loss [143] although the responsible mechanisms remain to be established. Resolving the contributions of HIV infection and AIDS, traditional osteoporosis risk factors, lifestyle factors, and ART to skeletal decline have been challenging [145, 147, 176]. In addition, because ART is used clinically as a combinatorial coformulation of multiple drug classes including protease inhibitors (PIs), nucleoside reverse transcriptase inhibitors (NRTIs), nonnucleoside reverse transcriptase inhibitors (NNRTIs), integrase inhibitors (IIs), and entry (fusion) inhibitors, with yet others in development, ascribing specific effects to specific drug classes has been difficult. This is further complicated by the fact that for each class of drug multiple variants exist that differ in chemical structure making for a very wide range of different formulations in different patients and between different studies.

Attempts to circumvent these confounders have involved *in vitro* or *in vivo* animal studies of ART; however the data has often been difficult to reconcile with clinical observations. PIs such as the ubiquitously used ritonavir have long been associated with bone loss in patients [176–181]; however when mice were treated with ritonavir in the absence of viral infection bone mass was found to be significantly improved rather than worsened [182]. Mechanistically, ritonavir was reported to inhibit osteoclast differentiation and abrogated bone resorption by disrupting the osteoclast cytoskeleton. Furthermore, ritonavir blunted parathyroid-hormone- (PTH-) induced osteoclastogenesis in mice and *in vitro* was found to suppress RANKL-induced NF-*κ*B and Akt activation, signaling pathways critical to osteoclast formation and function. By contrast in the same study indinavir, a PI of different chemical structure, was inert and exhibited none of the effects observed for ritonavir [182].

By contrast, in another study ritonavir and saquinavir (but not indinavir and nelfinavir) were reported to promote T-cell production of RANKL following exposure to soluble HIV-1 envelope glycoprotein gp120 *in vitro* [183].

NRTIs have also been associated with bone loss in humans [176, 177, 184]. Zidovudine (AZT) the first drug approved for treatment of HIV-1-infection is of the NRTI class and although seldom prescribed now has in fact been found to promote bone loss in mice and to stimulate osteoclast formation *in vitro* along with two other NRTIs Didanosine (DDI) and Lamivudine (3TC) [185, 186].

Although there is presently no agreement on the mechanisms of ART-induced bone loss* in vivo*, accumulating evidence suggests that to some degree all classes of ART have a negative influence on bone turnover [187, 188] and the magnitude of bone loss is greatest within the first 2 years of therapy [150, 189, 190].

Because all ART regimens act to rejuvenate immune function through a repopulation of T cells (and B cells), a process that begins immediately following ART-initiation, but often continues for up to two years [191], current research in our laboratory is investigating whether ART-induced bone loss is, in part, a consequence of immune reignition leading to a transitory inflammatory bone loss. The recovery of T cells (and B cells) in the setting of ART is complex and likely differs between patients, involving peripheral expansion of existing T-cell pools in some, and/or IL-7 mediated thymic reactivation in others [191, 192]. T-cell and B-cell homeostatic expansion is a process involving lymphocyte activation and proliferation, conditions propitious for production of inflammatory and osteoclastogenic cytokines including TNF*α* and RANKL. These mechanisms may significantly resemble the bone loss associated with animal models of postmenopausal osteoporosis (described later in detail) which too involve IL-7-driven thymic-dependent differentiation of bone-marrow-derived progenitors and thymic-independent peripheral expansion of mature T cells [193].

While bone loss in the context of HIV infection and ART is ultimately likely to be explained by a combination of multiple factors that likely differ significantly between different individual patients, the recent appreciation for how immune function controls basal bone turnover and the role of inflammation in driving bone loss is likely to provide a new perspective on skeletal degeneration in AIDS patients and ultimately lead to new therapies to combat bone loss, through modulation of the immunoskeletal interface.

### 6.4. Lymphocytes and Alveolar Bone Loss in Periodontal Infection

Periodontal infection is a major cause of tooth loss in adults and is characterized by inflammation of the supporting tissues of the teeth, resulting in resorption of alveolar bone as well as loss of the soft tissue attachment to the tooth [194].

B cells have been reported to limit bone resorption in periodontitis as evidenced by *in vivo* animal studies involving T-cell deficient nude rats or rats treated with anti-B cell antibody. Periodontal bone loss was assessed following inoculation with *Actinomyces viscosus* or *Bacteroides gingivalis* and revealed that anti-B-cell-antibody-treated rats had significantly less periodontal bone support, whereas no difference was found between normal, nude, and thymus-grafted rats. The study concluded that while permanent T lymphocyte deficiency did not interfere with periodontal disease development, acute moderate reduction in B cells predisposed the animals for aggravation of alveolar bone loss [195]. One explanation for this outcome is that B cells are necessary to control the growth of periodontal bacteria and indeed in this study 95% of the inoculated rats raised serum IgG or IgM antibody against one or more of the microorganisms to which they were exposed [195]. However, another possibility is that B-cell OPG production ameliorates alveolar bone loss. Interestingly, it has been reported that B cells activated in the presence of helper T cells (Th)1 cytokines were found to inhibit osteoclastogenesis while B cells activated in the presence of Th2 cytokines increased osteoclastogenesis [196].

Although T cells did not appear to play an important role in supporting periodontal bone loss in the Klausen et al. study [195], other investigations have indeed implicated T cells in periodontal bone loss [197]. In fact, periodontal tissues from human patients with periodontal infection have been reported to express significantly higher levels of RANKL protein and significantly lower levels of OPG. In this study RANKL was associated with lymphocytes and macrophages, the latter not being a typical source of RANKL, while OPG protein was associated with endothelial cells [198].

In another study unfractionated peripheral blood mononuclear cells from periodontal patients revealed a high degree of spontaneous T-cell-dependent osteoclast formation compared to controls and were consistent with overexpression of RANKL and TNF*α* by T cells. Furthermore, anti-RANKL and anti-TNF*α* antibodies significantly inhibited osteoclastogenesis suggesting that T cells support spontaneous osteoclastogenesis in periodontal disease via RANKL and TNF*α* overexpression [199].

Furthermore, high levels of serum IL-7 associated with peripheral blood B cells from periodontal patients have been suggested to be responsible for T-cell-dependent osteoclastogenesis [200]. Indeed we have reported that IL-7 is a potent inducer of RANKL production by human peripheral blood derived T cells [125] (discussed later in detail). Furthermore, bone loss may be exacerbated by TNF-related apoptosis-inducing ligand (TRAIL) production suppressing osteoblast formation [201].

In another study, double-color confocal microscopy revealed that less than 20% of B cells and T cells were found to express RANKL in healthy gingival tissues, while in diseased gingival tissues, more than 50% and 90% of T cells and B cells, respectively, expressed RANKL. As RANKL production by nonlymphoid cells was not observed, this study concluded that B and T lymphocytes are the primary sources of RANKL in the bone resorptive lesion of periodontal disease [169].

Taken together both T cells and B cells have been implicated in alveolar bone loss in periodontitis, likely due to enhanced RANKL production, as a consequence of immune activation and potentially exacerbated by diminished OPG production by B cells and secretion of inflammatory cytokines such as IL-6, IL-7, and TNF*α*.

### 6.5. Lymphocytes and Osteoclastic Bone Loss in Rheumatoid Arthritis

Rheumatoid arthritis is another inflammatory disease; however in contrast to periodontitis where inflammation is driven by bacterial infection, rheumatoid arthritis stems from an autoimmune condition leading to immune-mediated deterioration of cartilage and bone in the affected joints [202]. This is the result of a state of chronic inflammation that develops in the synovial membrane of affected joints and afflicts approximately 2 percent of the adult population causing chronic pain and fatigue, crippling, and loss of daily function and can cause permanent disability and increased mortality [203]. Rheumatoid arthritis not only causes destruction of cartilage but also disrupts systemic control of bone remodeling causing a systemic bone loss [204]. A major characteristic of rheumatoid arthritis is a dense lymphoid infiltration into the synovial membrane with high concentrations of activated T and B cells that drives not only initiation of the inflammatory state but is also in large measure responsible for the bone loss associated with rheumatoid arthritis [203, 205–208]. In rheumatoid arthritis activated T and B cells are potent inducers of osteoclastic bone resorption through direct RANKL secretion as well as through production of TNF*α*, a cytokine of key importance in the etiology of rheumatoid arthritis. In fact, transgenic overexpression of TNF*α* in mice leads to a robust inflammatory state characterized by bone and joint destruction that closely mimics that of human rheumatoid arthritis [209, 210]. TNF*α* ablation, by contrast, prevents both inflammation and bone loss in TNF*α* transgenic mice [82], and pharmacological TNF*α* inhibitors, such as TNF*α* receptor decoy receptors, are effective agents for amelioration of rheumatoid arthritis in human patients [84, 211–214].

Although bone erosions are ameliorated by TNF*α* inhibitors, it remains unclear whether this is a direct consequence of the beneficial action of these pharmaceuticals on TNF*α*-mediated inflammation (upstream of bone turnover) or whether there is a direct beneficial effect on bone formation and/or resorption or a combination of both mechanisms. Given studies by others and us in animal models, reduction of TNF*α* is expected to boost bone formation while simultaneously reducing bone resorption [90, 98, 113].

Interestingly, high levels of IL-7 have also been reported in rheumatoid arthritis and may contribute to the cycle of inflammation and/or bone loss [215–220]. Indeed, injections of IL-7 in arthritic mice cause expansion of T and B cells and increased levels of proinflammatory mediators and intensify arthritis severity and joint destruction. These actions were accompanied by increased Th1 and Th17 activity and suggest that IL-7 could be an important mediator in arthritic conditions and that targeting IL-7 or its receptor may represent a novel therapeutic strategy [221]. In contrast to IL-7 administration, antibody neutralization of the IL-7 receptor significantly reduced clinical arthritis severity in association with reduced radiographic joint damage [221].

Th17 cells are another major subset of T-helper cells and named for their expression of the cytokine IL-17 and have long been suggested to play a role in rheumatoid arthritis [222]. Levels of IL-17 in synovial fluids are reported to be significantly higher in rheumatoid arthritis patients than osteoarthritis patients, and anti-IL-17 antibody was shown to significantly inhibit osteoclast formation induced by culture media derived from rheumatoid arthritis synovial tissues. Furthermore, treatment of cocultures of mouse hemopoietic cells with primary osteoblasts stimulated with IL 17 induced osteoclast differentiation, which was completely inhibited by OPG. Expression of RANKL by osteoblasts in response to IL-17 was further confirmed *in vitro* suggesting that IL-17-induced osteoclastic bone loss is a consequence of RANKL expression by osteoblasts [223].

In another study IL-17 adenoviral overexpression in the knee joint of type II collagen-immunized mice (a murine model of rheumatoid arthritis) promoted osteoclastic bone erosions in cortical, subchondral, and trabecular bone along with expression of RANKL and its receptor RANK in the synovial infiltrate and at sites of focal bone erosions. IL-17 not only enhanced RANKL expression but also strongly upregulated the RANKL/OPG ratio in the synovium, while systemic OPG treatment prevented joint damage induced by local IL-17 overexpression. These findings suggest T cell IL-17 to be an important inducer of RANKL expression stimulating osteoclastogenesis and bone erosion in arthritis [224].

Consistent with these assertions antibody neutralization of IL-17 in a murine model of rheumatoid arthritis, the TNF*α* transgenic mouse, using IL-17 neutralizing antibody while only having minor effects on TNF*α*-induced inflammation, effectively reduced local and systemic bone loss by blocking osteoclast differentiation *in vivo*. These protective effects on bone erosions were consistent with a shift to bone-protective T-cell responses such as enhanced Th2 differentiation, IL-4 and IL-12 expressions, and increased regulatory T-cell (Treg) numbers. The data highlight IL-17 as a putative therapeutic target for ameliorating bone destruction associated with T-cell activation in rheumatoid arthritis [225].

## 7. Role of the Immunoskeletal Interface in Estrogen Deficiency-Induced Bone Loss

A cause-effect relationship between estrogen deficiency and postmenopausal osteoporosis has existed for over 70 years. Loss of estrogen at the time of the menopause results in an accelerated phase of bone loss impacting predominantly cancellous (trabecular) bone that declines rapidly over a 4–8-year period. Thereafter, this is replaced by a second slow phase of bone loss in which the rate of cancellous bone loss is reduced, but the rate of cortical bone loss is unchanged or increased. This bone loss continues indefinitely, mediated in large measure by the development of a state of secondary hyperparathyroidism [226].

One of the most surprising aspects to emerge in the past 15 years is the profound contribution of the immune system to bone loss in estrogen deficiency [15, 17, 227]. For more than a decade we have investigated the cascade of inflammatory cytokines that regulate the immune system and bone turnover in a murine model of estrogen deficiency, the ovariectomized mouse [15, 17, 43, 70, 77, 137, 193, 228–235]. Some of the key findings from these studies are presented in the following.

### 7.1. B Cells in Ovariectomy-Induced Bone Loss

The role of B cells in the bone loss associated with estrogen deficiency remains contentious. Almost 2 decades ago it was observed that ovariectomy in mice leads to a surge in B lymphopoiesis [236] and that estrogen administration can suppress B lymphopoiesis [237]. These studies lead to the suggestion that cells of the B lineage may contribute to ovariectomy-induced bone loss, and in 1997 a landmark paper demonstrated that the lymphopoietic cytokine IL-7 caused a potent rise in B lymphopoiesis when injected into mice, an event that was paralleled by a significant loss of BMD [238].

Interestingly, immature B cell populations expressing the marker B220 have been suggested to transdifferentiate along the osteoclast pathway *in vitro* [239] providing a potential enhanced source of osteoclast precursors and an explanation for a role of B-lineage cells in ovariectomy-induced bone loss. Although the potential for B220 cells to function as osteoclast precursors *in vitro* has been independently verified by us and others [240, 241], the role of such cells *in vivo* remains unclear.

After the discovery of RANKL as the key osteoclastogenic cytokine, expression of this factor by B-lineage cells (B220^+^ cells, which in the bone marrow represent multiple populations of early B-cell precursors, immature B cells, and mature B cells) has been reported to be more abundant in ovariectomized mice than in sham-operated mice [170]. RANKL from B cells isolated from the bone marrow of estrogen deficient postmenopausal women have been demonstrated to secrete RANKL [242], providing a plausible mechanism for a role of B cells in estrogen deficiency bone loss.

In an attempt to demonstrate a cause-effect relationship between B-lymphopoiesis and bone loss in estrogen deficiency we performed ovariectomy on B-cell KO mice and examined bone loss by DEXA and microcomputed tomography (*μ*CT). Surprisingly, after compensating for differences in basal bone mass between WT and B cell KO controls, ovariectomized B cell KO mice lost an identical percentage of bone mass to that of WT controls, excluding a role of mature B cells in bone loss associated with this model, although this KO retains early B-cell precursors that might contribute to the process [243].

Recently, however, to overcome the confounder of altered bone turnover in B-cell KO mice, Onal et al. made use of a state-of-the-art conditional B-cell RANKL KO mouse, to reevaluate the role of mature B cells in ovariectomy-induced bone loss. This high sensitivity model did indeed reveal a small contribution of mature B cells to ovariectomy-induced bone loss as mice lacking RANKL in B lymphocytes were partially protected from the increase in osteoclast numbers and bone loss caused by ovariectomy in cancellous bone, although not in cortical bone, in the conditional KO mice [122].

### 7.2. The Role of T Cells and IL-7 in Ovariectomy-Induced Bone Loss

A surprising finding over the last decade and a half has been the identification of a central role for T cells in the osteoclastic bone loss associated with ovariectomy in mice and recent translational data from clinical studies providing tantalizing support for a role of T cells in human postmenopausal bone loss.

In an attempt to further dissect the IL-7-induced bone loss reported by Miyaura et al., and ascribed to B cells [238], we examined the action of IL-7 in fractionated peripheral blood cells *in vitro* and made the surprising discovery that IL-7 potently promoted *in vitro* osteoclast formation but only in the presence of T cells. We further demonstrated that IL-7 stimulates the production of RANKL by T cells and that OPG could significantly, although not completely, repress IL-7-induced osteoclastogenesis [125]. Indeed, IL-7 is an established master regulator of T-cell homeostasis [244]; however these were the first studies to demonstrate that IL-7 is an inducer of T-cell RANKL production. Importantly, we were able to further demonstrate that ovariectomy enhances the production of IL-7 *in vivo* and that antibody directed neutralization of IL-7 prevented ovariectomy-induced bone loss in mice. In addition, IL-7 neutralization augmented bone formation suggesting that IL-7 was an osteoblast inhibitor and indeed IL-7 injection into mice inhibited bone formation *in vivo* suggesting that IL-7 may uncouple bone formation from bone resorption, exacerbating ovariectomy-induced bone loss [70].

Additional studies in mice revealed that IL-7-administration induces bone loss by a mechanism that involves production of RANKL, but also involved TNF*α* *ex vivo *[240]. In fact, *in vivo* IL-7 has multiple complex actions that impact almost every step in T-cell maturation, development, and function [244], and consistent with this concept we further demonstrated that in ovariectomy, IL-7 stimulated both thymic-dependent differentiation of bone-marrow-derived progenitors and thymic-independent, peripheral expansion of mature T cells. Almost 50% of the bone loss observed was through thymic-dependent effects of IL-7 as thymectomy decreases by half, the bone loss and stimulation of T lymphopoiesis induced by estrogen deficiency [193, 245]. The role of the thymus in postmenopausal osteoporosis in humans is presently unclear, given that at advanced age significant thymic atrophy has almost always taken place. However, studies in aged mice and rats have revealed that thymic regeneration may indeed occur, in part, in response to estrogen deficiency [246], and aged women have increased numbers of circulating recent thymic emigrants, an index of thymic output, relative to aged men [247].

How IL-7 is regulated is still unclear; however one study has reported that liver-derived insulin-like growth factor 1 (IGF-1) is permissive for ovariectomy-induced trabecular bone loss by modulation of the number of T cells and the expression of IL-7. In these studies IGF-1 KO mice were shown to be protected from ovariectomy-induced bone loss and T-cell expansion suggesting an action upstream of IL-7 [248].

Not all studies have reported a role for IL-7 in ovariectomy-induced bone loss. In fact, in contrast to the osteoclastogenic effects of IL-7 mediated through the immune system, it has been reported that IL-7 directly inhibits osteoclast formation *in vitro* [241]. In these studies IL-7 suppressed RANKL and M-CSF-induced osteoclast formation in murine bone marrow cultures derived from WT mice but not IL-7 receptor null mice. However, bone marrow from IL-7 receptor null mice generated significantly lower numbers of osteoclasts when treated with RANKL, vitamin D, or PTH showing complex effects of IL-7 [241]. The reasons for the different responses *in vitro* and *in vivo* are unclear, but may relate in part to the fact that IL-7-induced osteoclastogenesis *in vivo* occurs largely by a mechanism involving the stimulation of T-cell activation and expansion and production of RANKL and TNF*α*, while *in vitro* in the context of high doses of RANKL these effects are masked and a direct antiosteoclastogenic effect appears to be the dominant activity. Indeed, a similar effect is observed by IFN*γ* (discussed later in detail).

We have also reported that IL-7 is inhibitory to osteoblast differentiation [70] and may suppress the production of osteoclastogenic cytokines by these cells in coculture systems involving PTH and vitamin D-induced RANKL production.

In another model system, however, it was reported that IL-7 KO mice had increased osteoclasts and decreased trabecular bone volume compared with WT mice and lost similar amounts of trabecular bone following ovariectomy to that of WT animals. Interestingly, IL-7 KO mice were protected from cortical but not trabecular bone lost after ovariectomy [249]. Additional studies by the same group involving a model in which IL-7 was transgenically overexpressed using a 2.3-kb rat collagen 1a1 promoter to drive the expression of human IL-7 specifically in osteoblasts found increased trabecular bone volume *in vivo* by *μ*CT and decreased osteoclast formation in vitro. Surprisingly this bone phenotype was only evident in female mice. The authors hypothesized that overexpression of IL-7 in the bone marrow microenvironment may change the distribution and/or the differentiation of osteoclast progenitors by disturbing the balance of lineage development *in situ* [250].

In contrast to these studies a global IL-7 overexpressing transgenic mouse in which the IL-7 transgene was under control of an estrogen alpha promoter was reported to have a bone marrow cavity that was considerably expanded and with cortical bone showing focal osteolysis [251]. More recently in-depth analysis of this animal further revealed a specific phenotype characterized by an age-related loss of trabecular bone in both axial and long bones. Osteopenia was the result of an increased number of active osteoclasts on the surface of trabecular bone. The overexpression of IL-7 also created an osteoclastogenic bone marrow microenvironment that promoted the commitment of precursors towards the osteoclast lineage [252].

The conflicting reports for IL-7 actions by multiple independent groups of investigators reveal a complex action of this cytokine on bone homeostasis that remains to be fully understood.

Interestingly, bone disease occurs in over 70%–80% of patients with the B-cell cancer multiple myeloma and represents a significant source of morbidity and mortality [253]. Human myeloma cells have been reported to stimulate RANKL production by T cells, by a mechanism involving IL-7 and/or IL-6 [254]. This group has further shown a role for IL-7 in the suppressed osteoblast formation and differentiation induced by multiple myeloma cells from human patients [255]. Other studies have further investigated the mechanism for suppression of bone formation in multiple myeloma and report that increased levels of the transcriptional repressor growth factor independent 1 (Gfi1), a novel transcriptional repressor of the critical osteoblast transcription factor Runx2, may play an important role. Interestingly, Gfi1 induction was blocked by anti-TNF*α* and anti-IL-7 antibodies [256].

Taken together the data suggest that as in ovariectomy and rheumatoid arthritis IL-7 may play a critical role in the enhanced bone resorption and suppressed bone formation associated with multiple myeloma.

### 7.3. The Role of T-Cell TNF Production in Ovariectomy-Induced Bone Loss

TNF*α* has long been recognized as an important cytokine in postmenopausal osteoporosis [26, 42, 74, 75, 257]; however a surprising recent discovery was that T cells are the likely source of this cytokine. Support of a role for T-cell production of TNF*α* in ovariectomy-induced bone loss came from studies by our group demonstrating that ovariectomy-induced a significant expansion of TNF*α*-producing T cells in WT mice but failed to do so in T-cell deficient nude mice. The data suggested that ovariectomy caused an expansion of TNF*α* secreting T-cells, although without altering production of TNF*α* per cell. The critical role of TNF*α* production in ovariectomy-induced bone loss was further demonstrated in a study in which ovariectomy failed to induce bone loss in TNF*α* KO mice and mice deficient in the p55 (type I) TNF*α* receptor. By contrast, p75 (type II) TNF*α* receptor ablation failed to prevent ovariectomy-induced bone loss. Finally, adoptive transfer of T cells from TNF*α* KO mice into T-cell deficient mice revealed a key role for T-cell-specific TNF*α* production in the mechanism of ovariectomy-induced bone loss [228].

Interestingly, a recent study suggests that expansion of senescent CD4*⁺*CD28^−^ T cells may be the responsible TNF*α* secreting T-cell population involved in ovariectomy-induced bone loss as estrogen deficiency led to increased prevalence of TNF*α* secreting CD4^+^CD28^−^ T cells and even that could be reversed using the isoflavonoid daidzein [258].

The mechanism by which TNF*α* from T cells promotes osteoclast formation is not entirely clear, however it is established that TNF*α* amplifies the activity of endogenous RANKL as well as stimulating RANKL production by osteoblasts and their precursors [68, 259]. Interestingly, like B cells, T cells derived from estrogen deficient women have been demonstrated to express increased levels of RANKL [242] however a direct specific role for T-cell-derived RANKL in the mouse ovariectomy model has not been demonstrated. In fact, a recent study using a conditional RANKL KO mouse has demonstrated that ablation of RANKL in T cells failed to prevent or attenuate ovariectomy-induced bone loss [122]. These data suggest that although T cells have the capacity to secrete RANKL, it is predominantly TNF*α* that accounts for ovariectomy-induced bone loss.

Recent studies from our group show that the T-cell costimulatory molecule CD40L not only functions in the regulation of B-cell OPG production, but further mediates expansion of osteoblast precursors, promotes osteoblast proliferation, and differentiation and regulates osteoblast precursor production of M-CSF, RANKL, and OPG. Ovariectomy thus failed to promote bone loss and increase bone resorption in mice depleted of T cells or lacking CD40L, suggesting that cross-talk between T cells and osteoblast precursor mediated by CD40L plays a pivotal role in the disregulation of osteoblastogenesis and osteoclastogenesis induced by ovariectomy [235]. The data further suggest that RANKL production by osteoblasts may be the target of T-cell-derived TNF*α*.

Interestingly, a recent study further suggests that TNF*α* production from T cells promotes an increase in sclerostin, a potent Wnt-pathway antagonist and antiosteoblastic factor, that may contribute to the relatively reduced bone formation associated with estrogen deficiency. While sclerostin expression in WT mice was stimulated by ovariectomy and reversed by estrogen treatment as well as by pharmacological TNF*α* neutralization, T-cell deficient nude mice showed no response to ovariectomy [260].

## 8. IFN*γ*, a Key Mediator of Ovariectomy-Induced Bone Loss and T-Cell TNF*α* Production

Taken together the accumulated evidence suggests that estrogen deficiency leads to overexpression of IL-7 that culminates in T-cell activation and production of osteoclastogenic cytokines, predominantly TNF*α*. How T cells are activated in estrogen deficiency, however, turns out to be complex. CD4^+^ T cell activation requires two distinct signals, the first signal of which is generated by engagement of the T-cell receptor (TCR) on CD4^+^ helper T cells with major histocompatibility complex (MHC) class II (MHCII) bearing antigens on the surface of professional antigen presenting cells (APCs) including B cells, dendritic cells, and macrophages. This signal renders T cells inactive or anergic unless a second ‘‘costimulatory” signal is transmitted through association of the CD28 receptor on T cells, with B7 molecules (B7-1 (CD80) and B7-2 (CD86)), expressed on professional APCs. Once both signals have been received, the T cell becomes activated, undergoes clonal expansion, and produces characteristic cytokines depending on its ultimate differentiation program [261].

IL-7 is known to increase the sensitivity of T cells to otherwise weak antigenic stimuli, amplifying the antigenic response and driving up T-cell activation. IL-7 production thus acts to increase the magnitude of T-cell responses to otherwise weak antigens that are normally tolerogenic and that do not usually promote significant immune activation. IL-7 further increases immune activation by promoting production of IFN*γ*, a classic Th1 T-cell cytokine. IFN*γ* is a potent inducer of macrophage class II transactivator (CIITA) protein [262] a key transcription factor that upregulates MHCII expression on macrophages promoting antigenic responses.

Attesting to the importance of IFN*γ* in ovariectomy-induced bone loss we have further demonstrated that ovariectomy upregulates IFN*γ* that induces CIITA, increasing antigen presentation by macrophages, enhancing T-cell activation, and prolonging the lifespan of active T cells. As a consequence ovariectomy fails to induce bone loss in IFN*γ* receptor null mice. Attesting to the importance of antigen presentation in this system mice possessing a transgenic T-cell receptor that is responsive only to ovalbumin, a protein not endogenously present in mice, are protected from ovariectomy-induced bone loss, but undergo bone loss in response to estrogen deficiency following administration of ovalbumin [229].

The fact that ovariectomy was muted in the face of an inactive T-cell receptor strongly suggests that antigen presentation is a necessary component of ovariectomy-induced bone loss; however an intriguing question arises as to the nature of the antigen or antigens involved and whether a unique antigen is generated during estrogen deficiency that drives T-cell activation. In fact, because ovalbumin was found to support bone loss during estrogen deficiency the data would suggest that a specific type of antigen is not required and that any antigen capable of activating T cells is able to support bone resorption [229]. In addition, the capacity of IL-7 to reduce T-cell sensitivity to weak prevailing antigens coupled with the IFN*γ*-induced MHCII expression likely all converges to upregulate basal antigen presentation responses to endogenous foreign and self-antigens.

An interesting concept in immunology is that T-cell survival in the periphery is dependent on self-peptides that serve to maintain the longevity of mature T cells. This weak antigenic ‘‘tickling” does not induce T-cell activation or proliferation but simply helps to maintain the peripheral pool. In conditions where total T cell numbers are reduced, these self-ligands become overtly stimulatory and cause naive T cells to proliferate and undergo homeostatic expansion [263]. An intriguing possibility is that estrogen deficiency may promote T-cell expansion by a similar mechanism reliant on these physiological weak self-peptides that normally function in maintenance of T-cell homeostasis. An alternative explanation is that ovariectomy simply increases the sensitivity of T cells to foreign antigens including bacterial metabolites and cell membranes (LPSs), peptide products of digestion that are routinely absorbed in the gut and antigens inhaled into the lungs. Together, these foreign and self-antigens all conspire to sustain a weak basal antigenic activity that is necessary for the maintenance of T-cell peripheral pools [263–265].

Future studies remain to be conducted to definitively determine the types of antigens responsible for ovariectomy-induced bone loss.

Interestingly, IFN*γ* has further been reported to possess potent direct inhibitory actions on osteoclast formation by impeding the RANKL-RANK signaling pathway and inducing degradation of the RANK adapter protein, TRAF6 preventing RANKL-induced activation of NF-*κ*B and JNK [266]. Consistent with these studies IFN*γ* suppresses RANKL-induced cathepsin K expression in differentiating osteoclast precursors [267].

Given the need for IFN*γ* activity in promoting ovariectomy-induced bone loss *in vivo*, the data suggest that the direct inhibitory actions of IFN*γ* may compete with the proosteoclastogenic indirect actions. This was further investigated in T-cell null mice where systemic administration of IFN*γ* was found to cause bone loss in T-cell replete but not T-cell deficient mice. Thus under conditions of estrogen deficiency the net balance of IFN*γ* action was biased toward bone resorption in this study [232]. In another study administration of IFN*γ* *in vivo* was also reported to lead to bone loss and fails to ameliorate cyclosporin A-induced osteopenia [268]. Interestingly, in a study focusing largely on the effect of IFN*γ* on bone formation, IFN*γ* receptor KO mice were reported to reflect an osteoporotic phonotype due to reduced bone formation and elevated bone resorption [269].

The data suggesting antiosteoporotic actions of IFN*γ* conflict, however, with a number of clinical studies that have used IFN*γ* administration to treat excessive bone formation by upregulating bone resorption in conditions of osteopetrosis [270–272].

These different outcomes again reflect how subtle changes in experiment conditions may lead to very different results. While the actions of IFN*γ* appear to be contradictory between studies, it has been reported that early exposure to RANKL can prime osteoclast precursors to form in the presence of high levels of IFN*γ* using mechanisms independent of the signaling molecules signal transducers and activators of transcription (STAT)1 and TRAF6 [273], thus providing an explanation for how chronic IFN*γ* stimulation *in vivo* may overcome the inhibitory effects on osteoclast formation under inflammatory states.

## 9. TGF*β*, a Potent Inhibitor of Immune Activation and Ovariectomy-Induced Bone Loss

As discussed previously the role of TGF*β* in bone biology is extremely complex with both stimulatory and inhibitory effects on both osteoclastogenesis and osteoblastogenesis reported. Interestingly, in the context of estrogen deficiency and immune activation, TGF*β* appears to play an upstream protective role and represents a central mechanism by which estrogen protects the body against inflammatory bone loss.

We have shown that ovariectomy diminishes expression of TGF*β* in the bone microenvironment, while transgenic mice overexpressing a dominant-negative TGF*β* type II receptor, specifically in T cells, were completely insensitive to the bone-sparing effect of estrogen and as in the case of control WT mice ovariectomy led to production of IFN*γ*, T-cell activation, and T-cell TNF*α* production. Furthermore, overexpression of TGF*β* *in vivo* using a somatic gene therapy technique was found to prevent ovariectomy-induced bone loss. These findings suggest that stimulation of TGF*β* production in the bone marrow is a critical “upstream” mechanism by which estrogen prevents bone loss [137]. The reduction in TGF*β* signaling, associated with estrogen deficiency, may further serve to stimulate IL-7 production, thus intensifying the inflammatory response and driving up bone resorption [17].

The critical role of the immune system in ovariectomy-induced bone loss has been further validated in an independent study where estrogen deficiency failed to induce osteoporosis in immunocompromised bg-nu/nu-xid mice, a strain deficient in T, B, and NK cells [274].

In another study, however, ovariectomy was not found to alter the percentage of T lymphocytes in the bone marrow and spleen, and in fact small decreases were observed. In a comparison of bone mass in 3 strains of immunocompromised mice (T-cell deficient nude and TCR*α* KO mice and double T-cell and B-cell deficient recombination activating gene (RAG)2 KO mice) bone loss with ovariectomy was consistently observed in WT mice but was variably present in immunocompromised mice as measured by DEXA. In contrast, *μ*CT and histomorphometry showed similar trabecular bone loss after ovariectomy in all strains but found protection from cortical bone loss in nude and TCR*α* KO mice, but not in RAG2 KO mice [275]. The reason for the difference in outcomes between this study and that of others remains unclear.

## 10. TGF*β* and Regulatory T Cells (Tregs) in Bone Homeostasis

Tregs also known as suppressor T cells are a potent immunomodulatory T-cell subset that are critical for the prevention of spontaneous autoimmune disease by moderating inflammatory T-cell responses. Tregs further function to restore immune homeostasis after inflammatory responses in order to limit inflammation and prevent chronic inflammatory diseases [276].

Kim et al. found that Tregs inhibit osteoclast differentiation from peripheral blood mononuclear cells in a cytokine-dependent and cell-to-cell contact-independent manner and proposed that TGF*β* and IL-4 cytokine secreted by Th2 cells may be the key cytokines responsible for the suppressive function of Tregs [277]. Another recent study has implicated Tregs in the mechanism by which estrogen suppresses osteoclast differentiation and bone resorption through Treg production of IL-10 and TGF*β*1 [278].

Another mechanism by which Tregs maintain control of immune function is by secretion of Cytotoxic T-Lymphocyte Antigen 4 (CTLA4), an inhibitor that binds to CD80 and CD86 coreceptors on APC and blocking their association with CD28 on T cells, thus dulling inflammatory responses [279]. We have previously reported that one mechanism by which estrogen deficiency induces bone loss is by upregulating CD80 on dendritic cells. As an antagonist of CD80/CD28 costimulation we found that a pharmacological derivative of CTLA4, CTLA4-Ig, was able to prevent ovariectomy-induced bone loss by downmodulating the inflammatory cascade leading to T-cell activation and TNF*α* production and downmodulating osteoclastogenesis and bone resorption in mice *in vivo* [233].

Interestingly, Tregs have also been shown to directly inhibit osteoclast formation by CD11b monocytes treated with M-CSF and RANKL, as well as to suppress resorption of pits *in vitro* by mature osteoclasts. An important finding was that the key Treg cytokines TGF*β*, IL-4 and IL-10 were not essential to this inhibitory effect on osteoclastogenesis; however, direct antiosteoclastogenic effects of CTLA4 mediated on purified osteoclast precursors were documented. The data suggest that in addition to an anti-inflammatory role of CTLA4, this receptor may directly suppress osteoclastogenesis by binding to CD80/CD86 on mononuclear osteoclast precursors [280, 281].

## 11. The Th17 T-Cell Subset and Bone Loss in Estrogen Deficiency

Th17 cells are another subset of T-helper cells and named for their expression of the cytokine IL-17. To study the role of IL-17 in ovariectomy-induced bone loss Goswami et al. ovariectomized IL-17 receptor KO mice and quantified bone turnover. Surprisingly, the IL-17 receptor KO mice were found to be markedly more susceptible to ovariectomy-induced bone loss than sham controls. Although no changes in Th1, Th2, or Th17 cytokines were detected in serum, constitutively elevated leptin, a factor that regulates metabolism and satiety, but also bone metabolism, was further increased following ovariectomy and suggested as a putative mechanism to account for exaggerated bone loss [282].

In contrast to the IL-17 receptor KO, Tyagi et al. induced a functional block of IL-17 using neutralizing monoclonal antibody and found a complete prevention of ovariectomy-induced bone loss. Ovariectomy was further observed to lead to a significant expansion in Th17 CD4^+^ T cells and a dramatic elevation in the serum concentration of IL-17, events suppressed by administration of exogenous estrogen to mice. *In vitro*, IL-17 treatment was found to upregulate the expression of the receptor RANK on osteoclast precursors and to upregulate expression of TNF*α*, IL-6, and RANKL by osteoblasts. These effects were potently downmodulated by addition of estrogen to the cultures. These findings suggest that estrogen deficiency leads to increased differentiation of Th17 cells and induces bone loss by increasing proosteoclastogenic cytokines including TNF*α* and RANKL from osteoblasts [283].

## 12. Reactive Oxygen Species (ROS) and Immune Activation in Ovariectomy-Induced Bone Loss

Recently, increases in reactive oxygen species (ROS) have been implicated in the upstream actions of estrogen deficiency, aging and the bone loss associated with diabetes [233, 284, 285]. ROS have significant direct effects on the generation and survival of osteoclasts, osteoblasts, and osteocytes [284]. An important mediator of ROS in osteoblast progenitors is the ROS-activated FoxO transcription factors that converge on the bone anabolic Wnt signaling pathway, decoying *β*-catenin from binding to target genes necessary for osteoblast differentiation and decreasing osteoblastogenesis [284]. Ovariectomy not only alters the generation of ROS, but the antioxidant defense capacity of the cell is altered increasing sensitivity to the direct stimulatory effects of ROS on osteoclast formation and activity ([286, 287], Lean, 2004 #3124).

However, ROS also mediate potent actions on the immune system and have the capacity to indirectly promote osteoclastogenesis by altering the immunoskeletal interface. Superoxide promotes maturation of human dendritic cells and is reported to upregulate the costimulatory molecules CD80 and CD86 [288]. Indeed, we have further found that ROS induced by estrogen deficiency in mice upregulates CD80 on mouse dendritic cells [233]. Because CD80 is a key ratification signal for T-cell activation through its counterpart CD28 on the T cell [289], upregulation of CD80 facilitates antigen-dependent T-cell activation and hence promotes production of TNF*α* by T cells. Accordingly, bone loss is prevented by treatment of ovariectomized mice with either antioxidants or CTLA4-Ig, an inhibitor of CD80/CD28 costimulation [233].

## 13. Human Postmenopausal Osteoporosis and the Immunoskeletal Interphase

As the vast majority of studies have thus far been conducted using animal models of postmenopausal osteoporosis, these outcomes remain to be ratified in humans. In a number of cases animal studies have generated conflicting data, and consequently given the complexity and lack of uniformity among humans there are multiple complex outcomes that may be predicted.

One of the most interesting studies in humans addresses the production of RANKL by bone marrow cells in early postmenopausal women. In this study surface RANKL expression was quantified by two-color flow cytometry on isolated bone marrow mononuclear cells derived from premenopausal women, early postmenopausal women, and age-matched, estrogen-treated postmenopausal women. The surface concentration of RANKL per cell was increased in postmenopausal women compared to premenopausal women and estrogen-treated postmenopausal women by two- to threefold for MSCs, T cells, B cells, and total RANKL-expressing cells [242]. This study suggests that RANKL production by T cells and B cells may contribute to bone loss during estrogen deficiency in humans. Studies of human and animal lymphocytes do indeed suggest that both T and B cells have the innate capacity to secrete RANKL however a defined role for T-cell generated RANKL in ovariectomy-induced bone loss has not been demonstrated. In fact, the animal studies reported previously suggest that TNF*α* production by T cells is more important to the mechanism of ovariectomy-induced bone loss in mice.

Few human studies have been conducted to assess the role of T cells in postmenopausal osteoporosis but one recent clinical study reported that women with postmenopausal osteoporosis exhibit an increased T-cell activity and elevated production of TNF*α* and RANKL production compared to healthy postmenopausal controls [290]. These data provide enticing support to a role of T cells in the etiology of postmenopausal bone loss in humans as suggested by the animal models.

IFN*γ* is a cytokine with complex action *in vitro* and in animal models with both stimulatory [229, 232, 273] and inhibitory effects [266, 267, 269] being reported. 

In humans the weight of evidence is in favor of a net pro-osteoclastogenic activity as clinical studies have employed IFN*γ* to treat excessive bone formation by stimulating bone resorption in osteopetrosis [270–272].

Detailed clinical studies are now needed to establish the role of the immunoskeletal interface in human disease and the role of the inflammatory state in postmenopausal osteoporosis.

## 14. The Role of T Cells in the Bone Loss Associated with Hyperparathyroidism

Hyperparathyroidism is a condition whereby enhanced levels PTH lead to accelerated bone resorption and bone loss. High-turnover bone disease caused by secondary hyperparathyroidism is common in end-stage renal disease [291] and may contribute to bone loss in the slow phase of bone loss in postmenopausal women and in senile osteoporosis in aged men [226].

Primary hyperparathyroidism is associated with accelerated bone loss, osteopenia, and increased bone turnover and is an independent risk factor for fractures [292]. In animal models continuous infusion of PTH (cPTH) mimics primary and secondary hyperparathyroidism leading to osteoclastic bone resorption and bone loss. Paradoxically, when PTH is administered in an intermittent fashion (iPTH), mimicked in animals by a single daily injection, this pulsatile delivery of PTH leads to a relatively weak induction of bone resorption that is overcompensated for by a robust stimulation of bone formation leading to a net gain of bone mass [293]. In humans this anabolic effect of PTH has been exploited as a therapeutic strategy to improve bone mass in postmenopausal osteoporosis and other osteoporotic conditions [294].

Hyperparathyroidism and cPTH treatment increase bone turnover in trabecular and cortical bone, as evidenced by elevations in histomorphometric and biochemical markers of resorption and formation although mild hyperparathyroidism and cPTH injection in young mice may actually cause a modest increase in cancellous bone [292]. This bone loss is mediated, in part, through enhanced production of M-CSF [295] and RANKL by BMSC and osteoblasts, coupled with a decline in OPG production [296].

Over a decade ago an interesting report emerged describing the absence of a catabolic response to PTH in athymic T-cell deficient mice that underwent xenotransplantation of parathyroid gland fragments obtained from patients with primary or secondary hyperparathyroidism, and parathyroid cells maintained in culture from patients with secondary hyperparathyroidism. Despite extremely high plasma iPTH levels, hypercalcemia or hypophosphatemia was not observed, and no difference in active bone resorption surface or number of osteoclasts was seen by bone histomorphometry. The authors hypothesized that the characteristic deficit of T-cell function and of cytokine and growth factor production may protect nude mice with chronic hypersecretion of human PTH from hypercalcemia and bone lesions [297].

These data appeared to conflict however with an older study in which T-cell deficient nude mice were infused with human parathyroid hormone-related protein (PTHrP), a physiological ligand that binds to the same PTH receptor and has the same net biological activity as PTH. In this study serum calcium was significantly increased in nude mice infused with PTHrP compared with control nude mice, and static bone histomorphometry revealed increased osteoclast area, number of osteoclasts, and osteoclast perimeter in trabecular bone of lumbar vertebrae, as well as increasing bone formation in nude mice [298].

The reason for the discrepancies in response is unclear, however nude mice are a “leaky” phenotype that are known to recover T cells as they age due to extrathymic maturation. Furthermore, these animals have complex metabolism with delayed onset of puberty and rapidly lose fertility potentially resulting from changes in the estrogen responsiveness of tissues [275, 299, 300]. We have further reported that nude mice have increased BMD 4 weeks after birth but rapidly lose BMD relative to WT mice as they age, due to an imbalance in the RANKL/OPG ratio, a consequence of diminished B-cell OPG production [140].

Recently, we revisited the issue of T-cell-dependent PTH activity and confirmed that T cells play an essential permissive role in hyperparathyroidism as cPTH failed to induce osteoclast formation, bone resorption, and cortical bone loss in nude mice lacking T cells. We further demonstrated that T cells provide proliferative and survival cues to stromal cells and sensitize stromal cells to PTH through CD40L on activated T cells. Consequently, deletion of T cells or CD40L blunted the bone catabolic activity of PTH by decreasing bone marrow stromal cell number, RANKL/OPG production, and osteoclastogenic activity. To overcome some of the limitations of genetic T-cell KO models antibody-mediated deletion of T cells was further employed as an alternative model and further supported a need for T cells to mediate PTH-induced bone loss [301].

To investigate the role of direct PTH signaling in T cells during PTH-induced bone loss we used a mouse in which the PTH receptor had been conditionally silenced specifically in T cells using Cre/Lox technology. Using this strain a direct effect of PTH on T cells was shown to be necessary to support PTH-induced bone loss. Mechanistically, and similar to conditions of ovariectomy, PTH activation of the T-cell PTH receptor was shown to stimulate T-cell production of TNF*α*, suggesting a cause-effect relationship between this cytokine and cPTH-induced bone loss. To prove this the capacity of T cells to produce TNF*α* was conditionally ablated by reconstitution of T-cell deficient mice with T cells from TNF*α* KO mice, by adoptive transfer. The chimeric mouse with all sources of TNF*α* intact, except for that derived from T cells, was significantly protected from bone loss following PTH administration relative to T-cell deficient mice reconstituted with WT T cells [292].

Because CD40L expression is a feature of activated T cells, in a follow-up study we further demonstrated a role for antigen presentation in cPTH-induced bone loss and reported that inhibition of antigen presentation, through silencing of either class I or class II MHC-TCR interactions, prevented cortical bone loss induced by cPTH treatment *in vivo*. Finally, the costimulation inhibitor CTLA4-Ig was found to prevent iPTH-induced bone loss in WT mice demonstrating a need for normal APC activity to support iPTH-induced bone resorption [302].

Taken together the data suggest that T cells are essential participants in the bone loss associated with PTH and function in part by promoting T-cell production of TNF*α* and in part by sensitizing stromal cells to PTH through CD40/CD40L interactions.

## 15. The Role of T Cells in the Bone Anabolic Effect of PTH

Interestingly, T cells are not only pertinent to the bone loss associated with hyperparathyroidism, but also appear to be key mediators of the bone anabolic activity associated with iPTH administration.

Although T cells have long been implicated in the bone loss associated with hyperparathyroidism, there is no specific link between the anabolic activity of iPTH and T cells. Anabolic PTH is presently the only FDA approved anabolic therapy for fracture prevention in postmenopausal women [303]. Anabolic PTH improves bone mass and strength by promoting bone formation by increasing the number [304], activity, and longevity of osteoblasts through activation of preexisting quiescent bone lining cells [305], increased osteoblast proliferation [293, 306], and differentiation [293, 304, 306] and activation of survival signaling through attenuation of preosteoblast and osteoblast apoptosis [307–309].

An important mode of PTH action on bone formation involves regulation of the anabolic Wnt pathway in osteoclasts and their precursors culminating in the nuclear translocation of *β*-catenin. Wnt signaling is antagonized at the level of ligand binding to the Wnt receptors low-density lipoprotein receptor-related protein (LRP)5 and 6 by sclerostin, a protein synthesized in the bone microenvironment, in large measure by osteocytes [310]. Indeed a key mechanism of PTH activity is the downregulation of sclerostin leading to increased osteoblast sensitivity to prevailing Wnt ligands [311].

Interestingly, a key question that remains unclear is the nature and source of the Wnt ligands that support osteoblastogenesis in response to PTH.

T cells have long been recognized as a source of Wnt10b [312, 313], and we consequently investigated T cells as a source of this Wnt ligand under conditions of anabolic PTH treatment in mice. In fact, iPTH was found to significantly increase the production of Wnt10b by bone marrow CD8^+^ T cells leading to activation of canonical Wnt signaling in preosteoblasts. Demonstrating a key role of T cells in anabolic PTH action T cell null mice displayed diminished Wnt signaling in preosteoblasts and blunted osteoblastic commitment, proliferation, differentiation, and lifespan. These actions culminated in a diminished anabolic response in trabecular bone and a failure to increase bone strength. Furthermore, mice conditionally lacking Wnt10b production specifically in their T cells failed to induce an anabolic response to iPTH [314].

Further studies involving conditional silencing of the PTH receptor specifically in T cells were found to blunt the capacity of iPTH to induce T-cell production of Wnt10b, thus abrogating activation of Wnt signaling in osteoblasts, expansion of the osteoblastic pool, and increased BMD and trabecular bone volume in response to iPTH. These data thus revealed a direct action of PTH on the T cell leading to Wnto10b production [315].

Taken together that data demonstrated a key permissive role for T cells in the mechanism by which iPTH increases bone strength, suggesting that T-cell osteoblast crosstalk pathways may provide pharmacological targets for bone anabolism [314].

## 16. Conclusions

Over the past 2 decades phenomenal progress has been made in understanding how the immune system impacts and regulates the skeleton in physiological and pathological conditions through the immunoskeletal interface. Although most of the lessons learned have been in animal models and although the outcomes between studies have not always been consistent, tantalizing data from human studies has begun to translate and ratify some aspects of these findings in humans.

The existence of the immunoskeletal interface has wide repercussions for a range or disease conditions including inflection (periodontitis and HIV) and inflammation (rheumatoid arthritis, Crones disease, inflammaging, and estrogen deficiency, a condition that significantly resembles an inflammatory state).

Although bone loss is characteristic of aging, the immune system also undergoes significant changes during aging and immunosenescence is recognized as a major outcome. Contraction of T-cell receptor diversity leads to a diminished capacity of T cells to respond to pathogenic stimuli, and accumulation of autoreactive T cells that predispose to increased incidence of autoimmune disease is common. One of the most consistent characteristics of the aging immune system is the accumulation of CD28^−^ T cells that fail to respond productively to antigen presentation [316, 317]. An important question that remains to be answered is how these changes in the aging immune system impact the immunoskeletal interface and alter basal bone turnover, bone turnover associated with estrogen deficiency, and anabolic and catabolic bone turnover mediated by PTH.

A better understanding of these concepts may ultimately lead to novel approaches to the treatment of skeletal disease in multiple contexts involving, in part, modulators of immune function to treat downstream bone alterations. Indeed, the field of rheumatoid arthritis has been aggressive in adopting immunomodulatory biologic agents including TNF*α* inhibitors, T-cell costimulation suppressors, and B-cell depleting antibodies [213, 214, 318] to ameliorate inflammatory disease. Future therapeutic approaches may be able to incorporate some of these agents and pathways in some cases into new antiosteoporotic applications. The role of the NF-*κ*B pathway in bone formation and resorption as well as in inflammation further lends itself to exploitation for amelioration of bone disease.

Despite the progress made we have likely only scratched the “tip of the iceberg” concerning the functioning of the immunoskeletal interface in basal and pathological bone turnover, and many important discoveries likely await future elucidation.
